# Supplementary material for: Seismic reflections from a lithospheric suture zone below the Archaean Yilgarn Craton
Source: Nat Commun. 2021 Dec 13;12:7245. doi: 10.1038/s41467-021-27516-w (PMC8668964; doi:10.1038/s41467-021-27516-w)
Supplement: Supplementary file 1 — Supplementary Information [file 41467_2021_27516_MOESM1_ESM.pdf]

SUPPLEMENTARY INFORMATION

Seismic reflections from a lithospheric suture below the Archean Yilgarn Craton

Andrew J. Calvert[1] , Michael P. Doublier[2,3] , Samantha E. Sellars[1]

[1]Department of Earth Sciences, Simon Fraser University, Burnaby, British Columbia, V5A 1S6, Canada

[2]Mineral Systems Branch, Geoscience Australia, Symonston, ACT 2609, Australia

[3]Centre for Exploration Targeting, School of Earth and Environment, The University of Western Australia, 35 Stirling Highway, Crawley, WA 6009, Australia

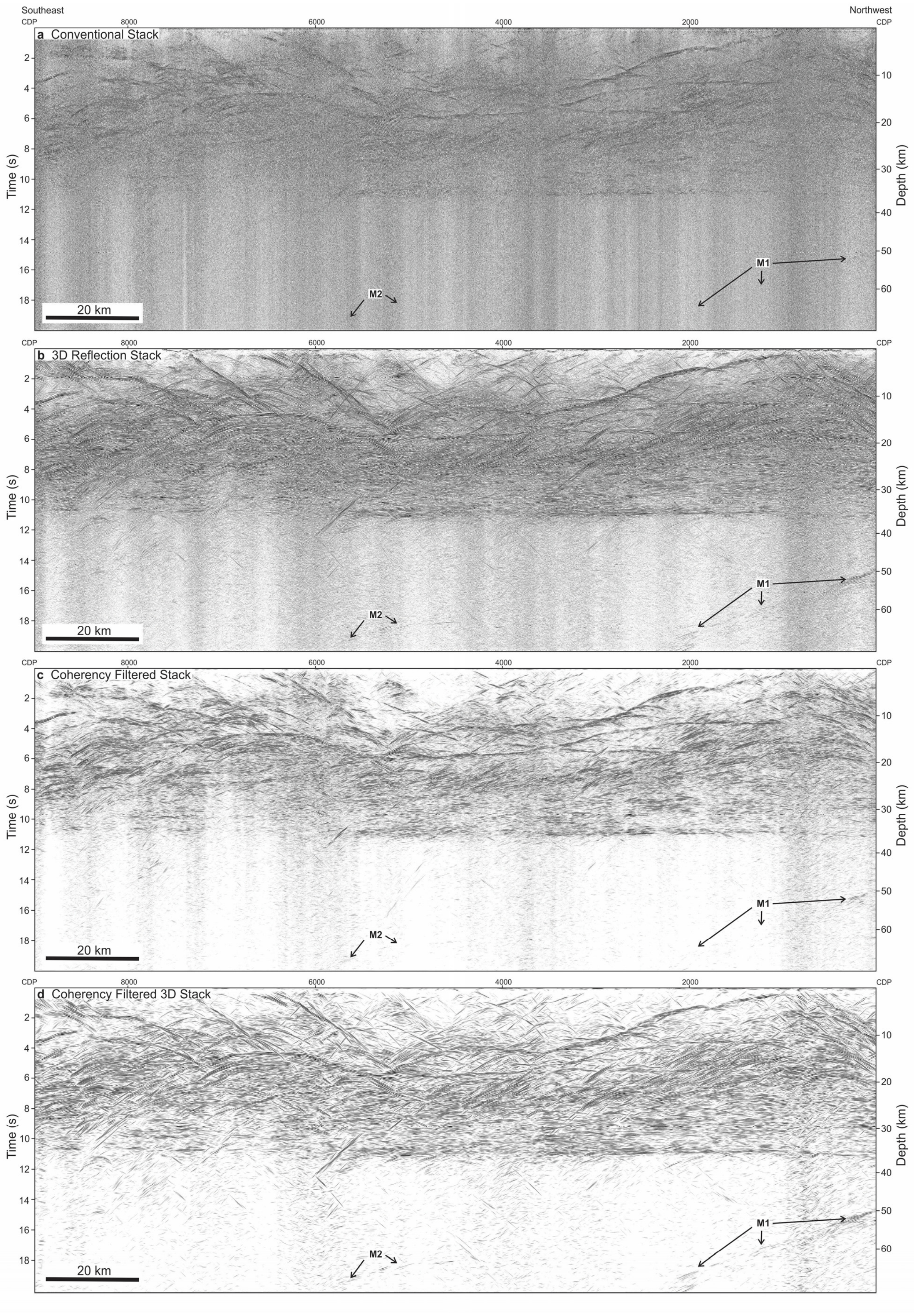
Southeast
Northwest
CDP
8000
6000
4000
2000
a Conventional Stack
b 3D Reflection Stack
c Coherency Filtered Stack
d Coherency Filtered 3D Stack
Time (s)
Depth (km)
M1
M2
20 km

Supplementary Fig. S1: Comparison of conventional processing of the northern half of line YU1 with a processing flow that identifies and stacks the most coherent reflections based on their 3D orientation. a Stack section obtained with conventional processing using 2D normal moveout correction. **b** Stack produced by summing reflection amplitudes over times corresponding to the most coherent reflection in 3D, as defined by its dip and strike values (See Methods section). **c** Coherency filtering of the conventional stack in A using summation over a 600 m aperture along the most coherent dip. **d** Coherency filtering of the 3D stack in B using the same 600 m aperture. All seismic sections are displayed after trace equalization using a 2-12 s time window.

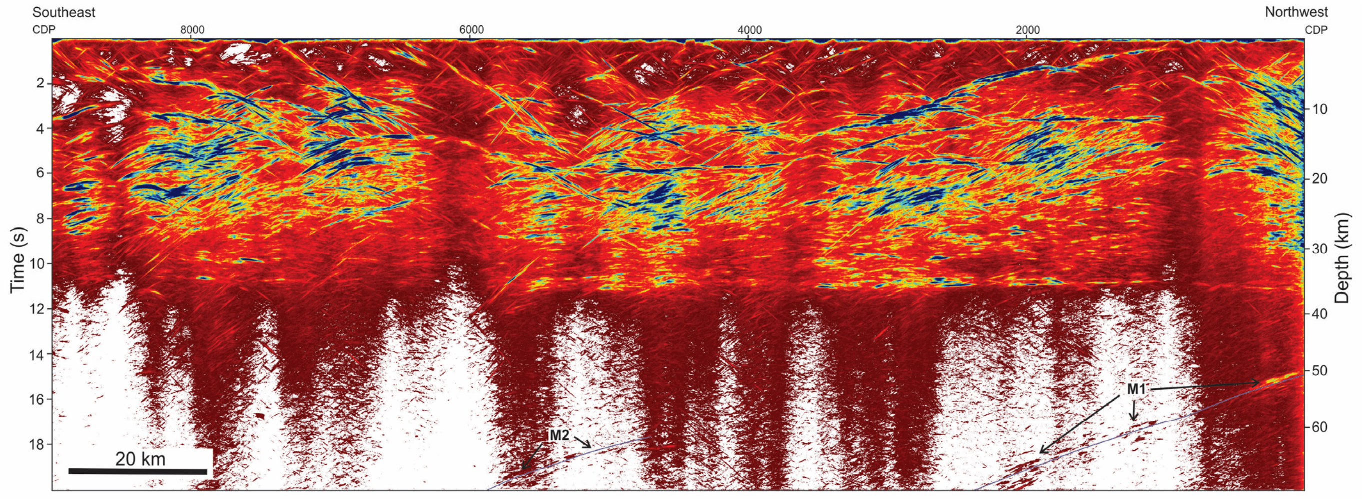

Supplementary Fig. S2: Semblance of the 3D reflection stack for the northern half of line YU1 over the Youanmi Terrane. The semblance values of crustal reflections, which are a measure of reflection coherency, are relatively high above the Moho at 11 s. Below 11 s, two packages of dipping reflections that correspond to reflectors within the upper mantle are highlighted, with solid lines indicating the best fitting planar reflectors with a strike of 095° from north. Other, more steeply dipping reflections below 11 s mostly originate within the crust due to their out-of-plane locations resulting in travel times greater than 11 s.

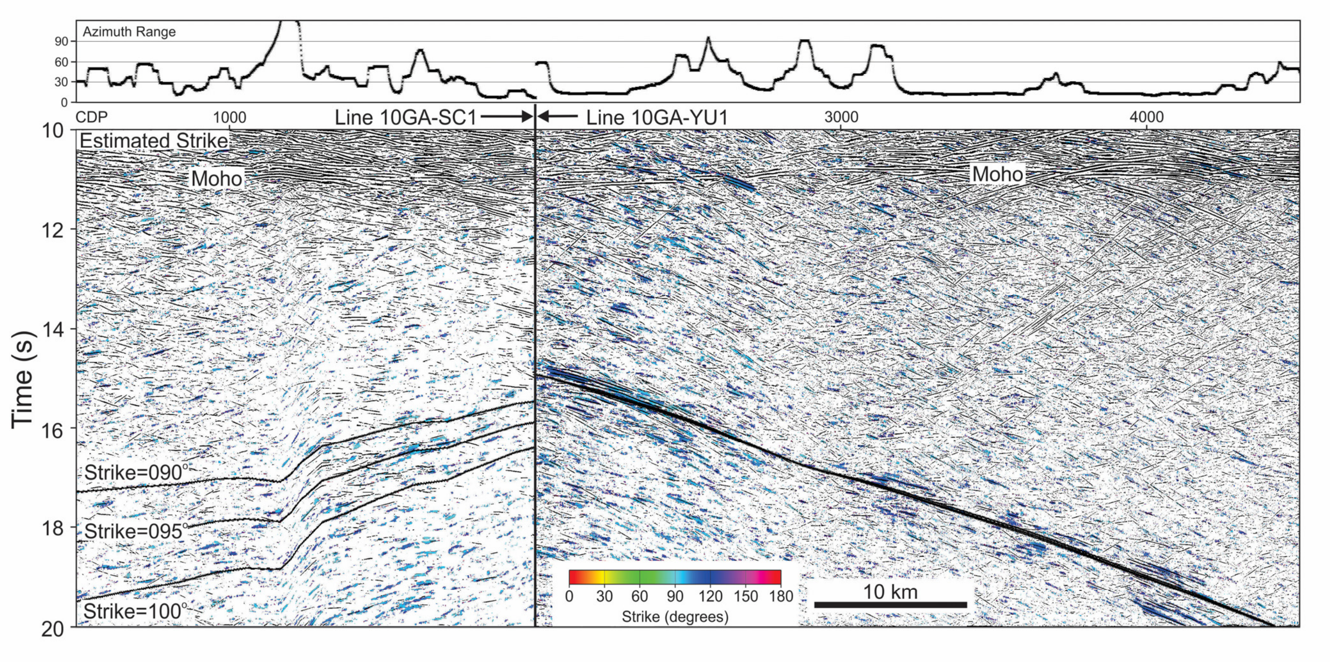

Supplementary Fig. S3: Unmigrated stacks of the east end of seismic line SC1 combined with the northern end of line YU1. The Moho corresponds to the downward termination of subhorizontal reflections at 11 s. The superimposed solid lines represent the zero offset travel times of three planar reflectors with dip/strike of 29°/090°, 32°/095°, and 36°/100° that were calculated for the crooked geometries of the two lines using a straight ray assumption and a root mean square average velocity of 6700 m s^{-1}. On line YU1, all travel times vary in a similar fashion to the arrival times of the band of mantle reflections On line SC1, short, discontinuous reflections that exhibit prestack strike azimuths of 90-120°, which are colored blue, are more common at times greater than 16 s, and likely originate from the mantle reflectors identified on line YU1. The onset of these mantle reflections lies between the travel times for reflectors with strike azimuths of 090° and 100°, and we consider a strike value of 095° to be a reasonable planar approximation to the geometry of the mantle reflector here.

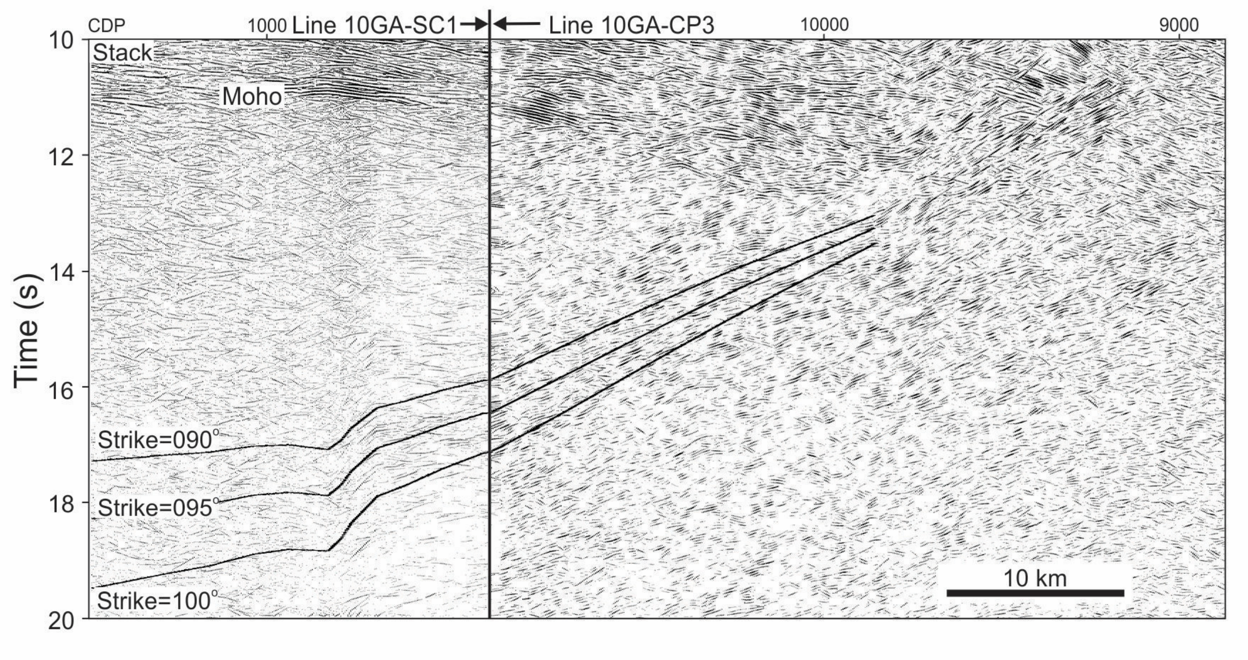

Supplementary Fig. S4: Unmigrated stacks of seismic lines SC1, west of the intersection point, and line CP3. On line SC1, the Moho corresponds to the downward termination of subhorizontal reflections at 11 s, and can be followed to 13.5 s near CDP 9000 on line CP3. The superimposed solid lines represent the zero offset travel times of three planar reflectors with dip/strike of 29°/090°, 32°/095°, and 36°/100° that were calculated for the crooked geometries of the two lines using a straight ray assumption and a root mean square average velocity of 6700 m s^{-1}. The different appearance of the two section is due the different processing applied (see Methods Section); line CP3 is shown after coherency enhancement.
